# Buruli ulcer in Australia: Evidence for a new endemic focus at Batemans Bay, New South Wales

**DOI:** 10.1371/journal.pntd.0012702

**Published:** 2024-12-13

**Authors:** Mehrab E Hossain, Caitlin Keighley, Andrew H. Buultjens, Jessica L. Porter, Paul D. R. Johnson, Timothy P. Stinear, Maria Globan, Caroline J. Lavender, Jake A. Lacey, Norelle L. Sherry, Anton Forsyth, Mark Formby, Ian Marr

**Affiliations:** 1 Department of Infectious Diseases and Microbiology, The Canberra Hospital, Australian Capital Territory, Australia; 2 Southern.IML Pathology, Sonic Healthcare, Wollongong, New South Wales, Australia; 3 Medical School, University of Wollongong, Wollongong, New South Wales, Australia; 4 Department of Microbiology and Immunology, University of Melbourne at the Peter Doherty Institute for Infection & Immunity, Victoria, Australia; 5 Department of Infectious Diseases and Immunology, Austin Health and University of Melbourne, Victoria, Australia; 6 World Health Organization Collaborating Centre for Mycobacterium ulcerans, Mycobacterium Reference Laboratory, Victorian Infectious Diseases Reference Laboratory, at the Peter Doherty Institute for Infection & Immunity, Melbourne Health, Victoria, Australia; 7 Microbiological Diagnostic Unit Public Health Laboratory, Department of Microbiology & Immunology, University of Melbourne at the Peter Doherty Institute for Infection & Immunity, Victoria, Australia; 8 Public Health Unit, Murrumbidgee and Southern New South Wales Local Health Districts, New South Wales, Australia; 9 Menzies School of Health, Darwin, Northern Territory, Australia; Stanford University, UNITED STATES OF AMERICA

## Abstract

We describe two locally acquired cases of *Mycobacterium ulcerans* infection (Buruli ulcer) in the town of Batemans Bay on the east coast of New South Wales (NSW), Australia, 150 km north of Eden, the only other place in NSW where Buruli ulcer has likely been locally acquired. Genomic analysis showed that the bacterial isolates from the cases were identical but belonged to a phylogenetically distinct *M. ulcerans* clade that was most closely related to the isolate from the earlier case in Eden to the south. It is proposed that Batemans Bay is a new endemic focus of human Buruli ulcer transmission.

## Author summary

*Mycobacterium ulcerans* causes Buruli ulcer, a progressive infection affecting skin and subcutaneous soft tissues. Although predominantly reported in central and west Africa, Australia, and Japan, new endemic regions for Buruli ulcer continue to be discovered. In Australia, the state of Victoria has seen a significant public health challenge with Buruli ulcer, where evidence points to zoonotic transmission from native possums to humans via mosquitoes. Recently, a new location of locally acquired Buruli ulcer has been identified in Batemans Bay, New South Wales (NSW), Australia. Genome sequencing of cases in this area has revealed a distinct *M. ulcerans* genotype, suggesting the pathogen is extant in NSW similar to the situation in Victoria. Surveys of possum excreta in Batemans Bay have confirmed the presence of *M. ulcerans*, indicating its establishment in a local wildlife reservoir in a new endemic region.

## Introduction

*Mycobacterium ulcerans* is responsible for causing a toxin-mediated, progressive, destructive infection of the skin and soft tissue known as Buruli ulcer [1,2]. This ulcer was likely first described in Uganda in 1897 by the missionary physician Sir Albert Cook, but the causative organism, *M. ulcerans*, was not formally identified as a new mycobacterial pathogen of humans until the 1940s in Victoria, Australia [3]. While predominantly reported in central and west Africa, Australia, and Japan, new Buruli ulcer endemic locations are still being encountered [4].

In Australia, Buruli ulcer has been increasingly reported in urban areas of Victoria, where the infection has become a significant public health issue following the initial appearance of a small number of localized coastal outbreaks [5]. The reservoir and mode of transmission of *M. ulcerans* have remained mysterious, but in Victoria, there is now evidence that Buruli ulcer is a zoonosis, with native possums as the key reservoir and mosquitoes as an important vector for transmission to humans [6]. Whether the zoonosis/insect transmission paradigm applies in other endemic regions, particularly Africa, has yet to be determined. Confirmation of high bacterial pathogen load with Buruli ulcer in possums in Victoria (as well as their excreta) has implications for further dissemination of this zoonosis in Australia [7].

Herein, we report a new site of locally acquired Buruli ulcer, in Batemans Bay, New South Wales (NSW), Australia. Genome sequencing and phylogeographic analysis revealed a distinct *M. ulcerans* genotype, that is consistent with the population structure of the pathogen in the region. The new cases we report here in Batemans Bay could be a harbinger of a disease expansion in NSW similar to Victoria. Additionally, we conducted a survey of possum excreta in a small area near the reported cases to determine the presence of *M. ulcerans* in the local possum population.

### Case 1

A 94-year-old Caucasian male reported that his left fourth finger became entrapped between the folding legs of an outdoor table at his home in Batemans Bay in November 2020. There was no history of travel outside his local area for at least four years. This trauma resulted in pain and swelling to the fourth proximal phalanx over four weeks. He did not recall a previous skin lesion or insect bite at the site. By mid-to-late December 2020, the wound progressively opened on the dorsal aspect of the fourth digit. Seeking treatment at the local hospital, intravenous cefazolin was administered for five days. Although he remained systemically well, due to ongoing progression and expansion of the skin lesion, he was transferred to Canberra Health Services in the Australian Capital Territory (ACT), Australia, for assessment.

**Fig 1:**
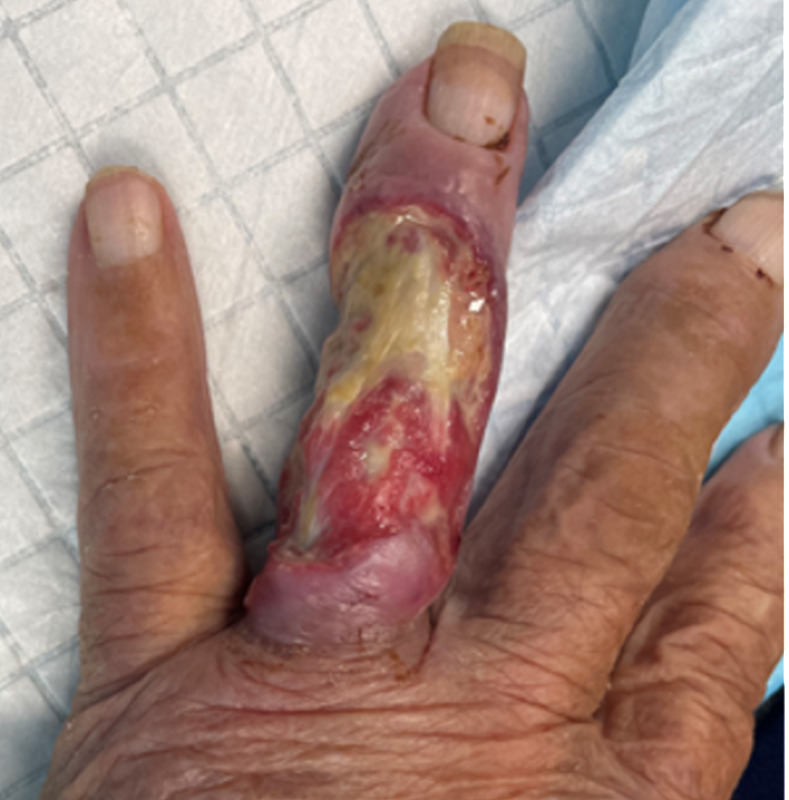
Dorsal necrotic ulcer to fourth digit.

**Fig 2:**
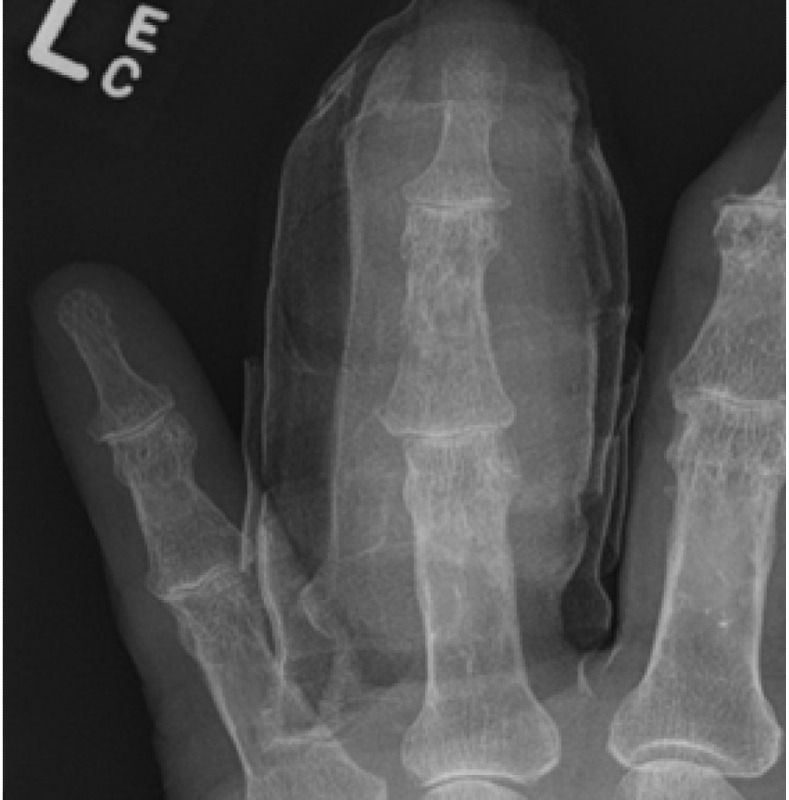
X-ray left fourth digit proximal and middle phalanx, showing focal bony lysis and cortical erosion.

Microscopic examination and bacterial culture of the initial superficial wound swab identified a mixed growth, including multiple *Pseudomonas* species and coliforms. A subsequent surgical debridement was performed (Fig 1) during which sampled tissue was subjected to further microbiological testing, including being sent for acid-fast bacilli (AFB) stain and mycobacterial culture. An X-ray of the affected finger confirmed changes consistent with osteomyelitis of the middle and proximal phalanges (Fig 2).

Before results could be confirmed, the patient underwent terminalization of the left fourth finger. Histopathology of the original debrided surgical specimen revealed the presence of necrotic tissue with Ziehl-Neelsen (ZN) stain-positive bacilli. *Mycobacterium ulcerans* was identified by polymerase chain reaction (PCR) performed on soft tissue from the finger [8]. Following successful source control and the absence of clinical evidence of residual *M. ulcerans* infection, antibiotic therapy was deemed unnecessary. The patient subsequently experienced a favorable recovery at the site of the proximal wound after the amputation.

### Case 2

**Fig 3:**
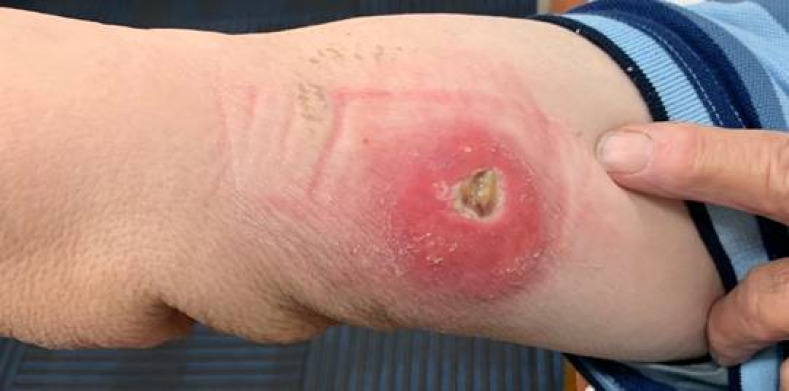
Dry, small ulcer on the inner aspect of the right upper arm.

A 71-year-old previously well Caucasian male noted a mosquito bite on the inner aspect of his right upper arm in mid-May 2023. This occurred while sitting in an armchair in his living room at home in Batemans Bay. He did not recall any interstate or international travel for at least three years. A red mark on the inner side of his right upper arm evolved into a small ulcer by late June, suggesting an incubation period of four to five weeks (Fig 3). Despite being prescribed courses of oral cephalexin and dicloxacillin, aimed at treating methicillin-susceptible *Staphylococcus aureus* identified from a superficial swab, there was no improvement.

**Fig 4:**
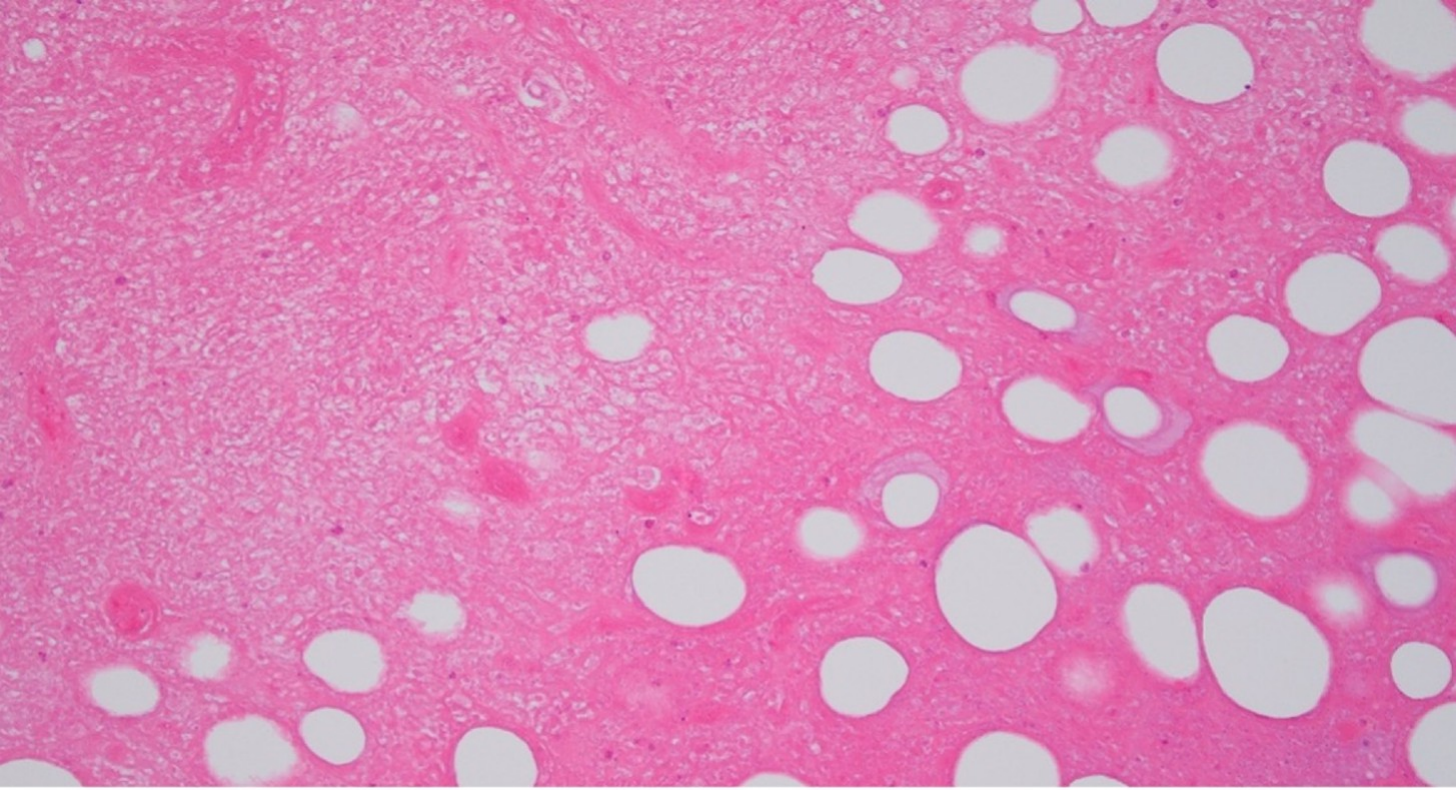
Hematoxylin and eosin stains (original magnification ×200) from tissue biopsy of lesion, showing fat necrosis.

**Fig 5:**
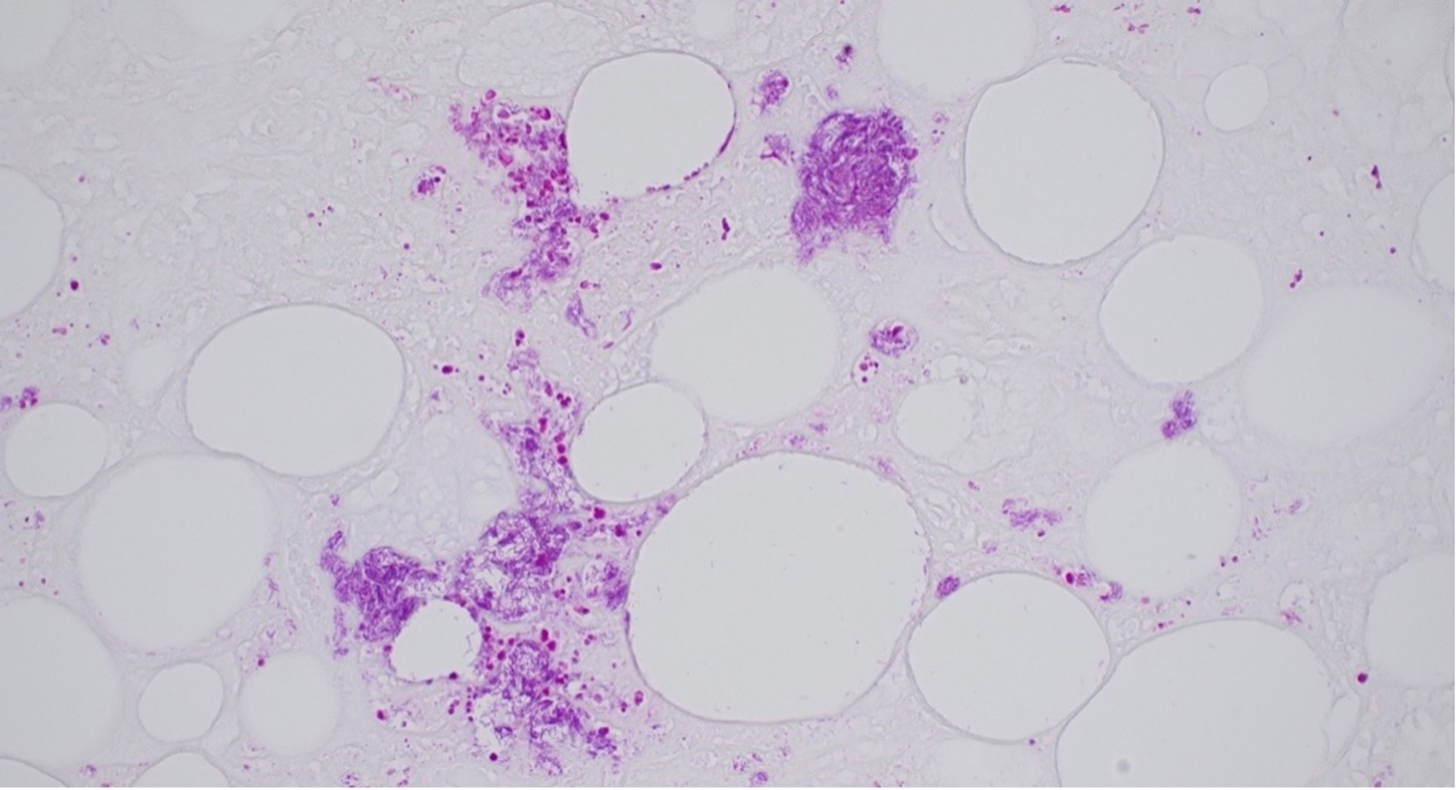
Ziehl-Neelsen stain (original magnification ×400) demonstrating acid-fast bacilli in the wound biopsy.

By early July, the ulcer had expanded to four by four centimeters. While it was tender to touch, it was otherwise not painful, and he did not require analgesia. There was a rubbery texture underlying the surface. The patient remained systemically well. He was referred to a general surgeon, and a biopsy in mid-July 2023 demonstrated extensive fat necrosis with numerous acid-fast bacilli (Figs 4 and 5). The 16S and internal transcribed spacer (ITS) region amplification revealed mycobacterial DNA but was unable to distinguish between *M. marinum and M. ulcerans*. This was subsequently confirmed to be *M. ulcerans* via a targeted IS*2404* PCR assay performed on a swab taken from the ulcer [8].

He was commenced on rifampicin 600 mg daily and clarithromycin 500 mg twice daily and experienced improvement of the ulcer over the subsequent two months. By the three-month mark, the lesion had largely resolved.

## Methods

### Ethics statement

Clinical samples from Buruli ulcer patient lesions were tested under Institutional Review Board (IRB) exemption for the use of de-identified pathology specimens, as per section 3.2.6 of the Australian National Health and Medical Research Council, National Statement on Ethical Conduct in Human Research (2023): “Where human biospecimens were obtained for clinical purposes and have been retained by an accredited clinical pathology service, the biospecimens may be used for research purposes if the identity of the donor is not necessary for the activity”.

### Mycobacterial detection

Tissue sample from case 1 and a swab from case 2 were collected for *M. ulcerans* PCR (DNA extraction followed by PCR detecting the IS*2404* amplicon, specific for *M. ulcerans*).

Tissue sample from case 1 was incubated in Mycobacteria Growth Indicator Tubes (MGIT) containing modified Middlebrook 7H9 Broth base in the liquid media culture, BD BACTEC MGIT 960 automated system (Becton, Dickinson, Sparks, MD, USA). Growth was observed at approximately six weeks. This was then referred to the Victorian Infectious Diseases Reference Laboratory (VIDRL) for identification and DNA extraction, and whole genome sequencing (WGS) was performed at the Microbiological Diagnostic Unit (MDU) Public Health Laboratory, Victoria.

For case 2, the swab was cultured on Brown and Buckle culture media and incubated at 31°C. On detection of growth, the isolate was confirmed as *M. ulcerans* by PCR and DNA was referred for WGS as for case 1.

### Genomic sequencing

Genomic DNA was prepared from *M. ulcerans* cultures and sequenced using Illumina paired end whole genome sequencing technology. The resulting reads were combined with a set of 44 publicly available clinical isolates that were chosen to depict the main components in the previously defined population structure of *M. ulcerans* in south-eastern Australia (S1 Table) [9]. Snippy (v4.4.5) was used to map sequence read data against a finished *M. ulcerans* reference chromosome, isolated from a south-eastern Australian Buruli ulcer case (JKD8049; GenBank accession NZ_CP085200.1; https://github.com/tseemann/snippy). An alignment of core genome single nucleotide polymorphisms (SNP) was derived from mapped reads and used to estimate a maximum likelihood phylogeny using the GTR model of nucleotide substitution with FastTree (v.2.1.10) [10]. The R packages phytools (v.1.0–1) [11] and mapdata (v.2.3.1) [12] were used to align tree tips against a base map to visualize the geographical origins of the clinical isolates.

### Possum excreta survey

Australian native possums are a well-established wildlife reservoirs of *M. ulcerans*. Possums develop Buruli ulcer lesions similar to human lesions, but unlike humans, they also shed the bacteria in their excreta [7]. Field surveys of possum excreta for the presence of *M. ulcerans* are a convenient means to assess the risk of Buruli ulcer transmission to humans [7]. We conducted a possum excreta survey over a two-day period in December 2023. The survey covered approximately 1.5 km by 1.5 km near the location of the reported cases. The survey involved roadside collection of possum excreta, following a pre-determined 200-meter grid pattern along residential streets. Excreta was not found at all pre-determined sampling locations; however, it was collected from 27 locations, providing a good representation of the survey area.

## Results

### Confirmation of *M. ulcerans*

The specimens from cases 1 and 2, collected for *M. ulcerans* PCR, returned with cycle threshold values of 28 and 18, respectively. Positive cultures on solid culture medium and in broth (MGIT tubes) were referred to VIDRL, and to MDU Public Health Laboratory for WGS.

### Comparative genomics

**Fig 6:**
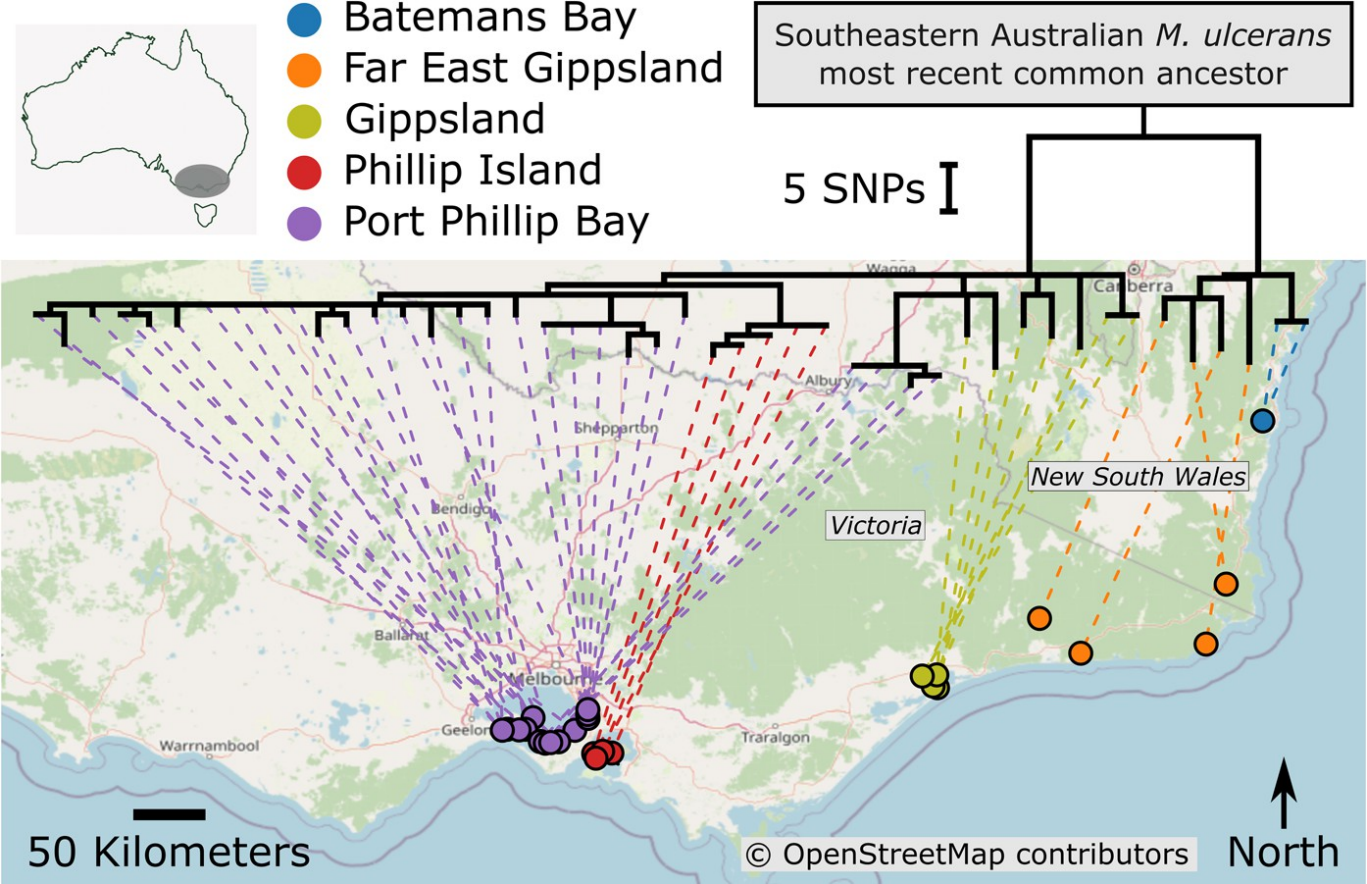
Phylogeographic analysis of Batemans Bay isolates in the context of the population structure of *M. ulcerans* in south-eastern Australia. A maximum likelihood phylogenomic tree of *M. ulcerans* isolates was estimated using core genome SNPs, with branch lengths corresponding to SNP distances. The tree illustrates the clustering of isolates, with isolates from Batemans Bay and Far East Gippsland forming a distinct cluster. The lineage harboring the Batemans Bay and Far East Gippsland isolates traces back to the south-eastern Australian *M. ulcerans* most recent common ancestor, before branching out towards isolates from the more western localities of Gippsland, Phillip Island, and the Port Phillip Bay area. Link to the Open Street Map basemap: https://www.openstreetmap.org/#map=7/-36.752/144.278; link to the Open Street Map copyright and license information: https://www.openstreetmap.org/copyright.

The alignment of DNA sequence reads obtained from each of the patient isolates to the JKD8049 reference chromosome facilitated the identification of 130 core genome single nucleotide polymorphisms (SNP). These SNPs were then used to infer a maximum likelihood phylogenomic tree. The core genome tree distinctly delineated the Batemans Bay isolates, setting them apart from the clinical isolates in Victoria in their own clade (Fig 6). Nevertheless, the Batemans Bay isolates exhibited a closer phylogenomic relationship with those from Far East Gippsland, the region geographically nearest to Batemans Bay (around 150 km away). Notably, the isolates from Batemans Bay and Far East Gippsland are situated on a distinct branch of the phylogenetic tree, which merges at the most recent common ancestor of *M. ulcerans* in south-eastern Australia (Fig 6). From this root point, the lineage harboring the Batemans Bay and Far East Gippsland isolates splits, leading away from isolates from the western endemic areas such as Gippsland, Phillip Island, and the Port Phillip Bay region. In previous research we used phylodynamic modelling and established a molecular clock of 0.422 SNPs/genome/year for *M. ulcerans* from southeastern Australia [9]. Applying this clock to the two Batemans Bay *M. ulcerans* genomes and the observation that these genomes are separated by one SNP from the east Gippsland most recent common ancestor (MRCA) that emerged in approximately 1980, we inferred that the Batemans Bay ancestor likely first emerged in the late 1970s (S1 Fig).

### Possum excreta survey

Out of the 27 samples collected for a possum excreta survey within a small area near the location of the reported cases, two samples tested positive for IS*2404* by qPCR.

## Discussion

Since the initial discovery of *Mycobacterium ulcerans* in the 1940s, new regions with local transmission of Buruli ulcer have continued to emerge [13]. We describe the first locally acquired cases of Buruli ulcer in Batemans Bay, NSW, Australia. The appearance of this phylogenomically divergent lineage, causing locally acquired infections, underlines the propensity for *M. ulcerans* to become established in new geographically separate possum populations as has been well documented in Victoria, Australia. The risk for further spread along coastal NSW is significant.

Both cases described above reside in distinct pockets of bushland within Batemans Bay, which is on the southeastern coast of Australia, approximately 520 km northeast of Melbourne and 110 km southeast of Canberra. The local permanent population at the latest census was 8,581 [14], but the region attracts many more national and international tourists annually. As a coastal town that sits at the mouth of the Clyde River, it is surrounded by densely forested native vegetation. With the convergence of native forest, saltwater, and freshwater, the area supports a broad range of aquatic and land-based wildlife, including possums. Studies have explored the possibility of mammals serving as a reservoir for Buruli ulcer [15,16]. *M. ulcerans* has been detected in the scat of possums at locations where human cases of the disease have been reported previously [16]. Both ringtail and brushtail possums are susceptible to natural infections, which result in skin ulcerations, and both may excrete *M. ulcerans* in high concentration in their feces [17]. The detection of positive possum excreta samples from Batemans Bay establishes beyond doubt that *M. ulcerans* is present in local possums. Larger, more systematic surveys will be required to examine the extent of possum involvement and to monitor change over time.

The phylogenomic separation of isolates from Port Phillip Bay, Phillip Island, and Gippsland from those of Far East Gippsland and Batemans Bay is traced back to the south-eastern Australian *M. ulcerans* most recent common ancestor. This deep divergence indicates a significant historical split and diversification in the pathogen population in the eastern part of south-eastern Australia [9]. This split has subsequently led to the emergence of the mycobacterial population in Batemans Bay which is distinct from the lineage prevalent in the most endemic areas of Victoria. The close genomic relatedness of the Batemans Bay isolates to those from Far East Gippsland suggests a pattern of spatial correlation, wherein isolates from geographically proximate regions are more closely related at the genomic level. This observed ’distance decay’ effect supports the hypothesis that the *M. ulcerans* in Batemans Bay has arisen from a local bacterial population, making it likely that *M. ulcerans* has been extant in the region for some time. Why Buruli ulcer cases have appeared in the last three years is a question that remains to be answered, but by extrapolation from observations in Victoria, expansion of the bacterial burden in a local possum population is a predictor of increased Buruli ulcer risk in humans [18].

Various transmission routes for *Mycobacterium ulcerans* have been proposed, with direct contact with contaminated environments suggested as one likely mode of infection in regions such as Africa and Australia [16,19,20]. In Case 1, the patient sustained blunt trauma prior to the development of Buruli ulcer and associated osteomyelitis. Given the detection of *M. ulcerans* in possum excreta in Batemans Bay and the potential for environmental contamination, it is conceivable that microabrasions from contact with the folding legs of an outdoor table facilitated pathogen entry. While the patient did not observe possum feces, the possibility of *M. ulcerans* contamination of the outdoor table remains. However, this explanation does not fit well with experimental evidence in hairless guinea pigs, where the application of cultured *M. ulcerans* cells, in high concentrations to experimentally created abrasions consistently failed to establish an infection [21]. Similar experiments performed in a mouse tail model of *M. ulcerans* infection showed the same failure to infect via direct contact [22]. Alternatively, there are reports that blunt trauma can trigger the sudden clinical manifestation of an infection that may have already been present [23].

In case 2, a mosquito bite was thought to precede the development of Buruli ulcer. In Victoria, mosquitoes are now thought to be the predominant mode of transmission to humans [18,24]. Also in Victoria, a mean incubation period for *Mycobacterium ulcerans* infection of 4.5–5 months has been established, with a range of 32 to 277 days [25,26]. Both of our patients experienced a relatively short incubation period of only a few weeks. The reasons for the wide variation in the incubation period remain unclear. In individual cases, there is the possibility of mistaken recall given the known long incubation period and the high frequency of more recent bites and minor trauma.

The possum excreta survey had limitations, including the small number of samples collected and the restricted geographic scope, which was confined to a single time point. While the detection of positive samples confirms the presence of *Mycobacterium ulcerans* in the local possum population, the limited size and scope of the survey constrain the ability to draw broader conclusions about the distribution and prevalence of the organism. Future studies incorporating larger survey areas and multiple time points would be valuable for gaining a more comprehensive understanding of the extent and distribution of affected possum populations in the region.

Given the many similarities in wildlife composition and insect presence between coastal Victoria and Eden and Batemans Bay in NSW, it is likely that NSW Public Health authorities are now facing progressive expansion of Buruli ulcer endemic areas and an increase in human infections, just as has been in Victoria. Among many unanswered questions in both Victoria and NSW is why new endemic foci of Buruli ulcer transmission appear discontinuously, sometimes hundreds of kilometers away from existing foci. The relatively long branch length of the Batemans Bay *M. ulcerans* genomes, revealed by the phylogenomic analysis, is consistent with—as yet—unsampled genetically diverse *M. ulcerans* present on other areas of Australia (Fig 6). That is, while Buruli ulcer occurrence in humans is discontinuous in its distribution, it is possible that *M. ulcerans* is more widely spread, harbored by wildlife reservoirs across Australia. Further research is needed to uncover the pathways of transmission that contribute to these sporadic outbreaks, which will be essential for controlling the disease’s spread and protecting communities.

## Supporting information

S1 Table *M. ulcerans* isolates and their respective sequencing reactions. (DOCX)

S1 Fig Estimation of the emergence date of the Batemans Bay *M. ulcerans* genotype. Shown is the relevant region of the *M. ulcerans* core-genome phylogeny and the internal tree nodes with divergence time estimations.
(DOCX)
