# Supplementary material for: Buruli ulcer in Australia: Evidence for a new endemic focus at Batemans Bay, New South Wales
Source: PLoS Negl Trop Dis. 2024 Dec 13;18(12):e0012702. doi: 10.1371/journal.pntd.0012702 (PMC11676496; doi:10.1371/journal.pntd.0012702)
Supplement: S1 Table — (DOCX) [file pntd.0012702.s001.docx]

| **Isolate ID** | **Short Read Archive** | **Region** | **Locality** | **Year** |
| --- | --- | --- | --- | --- |
| AUSMDU00063286 | SAMN42382909 | Batemans Bay | Batemans Bay | 2021 |
| AUSMDU00093002 | SAMN42382910 | Batemans Bay | Batemans Bay | 2023 |
| DMG1701435 | SRR6346206 | Far East Gippsland | Far East Gippsland | 2002 |
| DMG1701442 | SRR6346230 | Gippsland | Gippsland | 2002 |
| DMG1701365 | SRR6346234 | Port Phillip Bay | Bellarine Peninsula | 2015 |
| DMG1701366 | SRR6346235 | Port Phillip Bay | South Mornington Peninsula | 2015 |
| DMG1701361 | SRR6346238 | Port Phillip Bay | South Mornington Peninsula | 2015 |
| DMG1701362 | SRR6346239 | Port Phillip Bay | South Mornington Peninsula | 2015 |
| DMG1701359 | SRR6346240 | Port Phillip Bay | Bellarine Peninsula | 2015 |
| DMG1701360 | SRR6346241 | Port Phillip Bay | South Mornington Peninsula | 2015 |
| DMG1701368 | SRR6346243 | Port Phillip Bay | Bellarine Peninsula | 2015 |
| DMG1701459 | SRR6346244 | Phillip Island | Phillip Island | 2007 |
| DMG1701357 | SRR6346246 | Port Phillip Bay | South Mornington Peninsula | 2015 |
| DMG1701358 | SRR6346247 | Port Phillip Bay | South Mornington Peninsula | 2015 |
| DMG1701349 | SRR6346254 | Port Phillip Bay | South Mornington Peninsula | 2015 |
| DMG1701420 | SRR6346276 | Gippsland | Gippsland | 2009 |
| DMG1701423 | SRR6346279 | Port Phillip Bay | North Mornington Peninsula | 2009 |
| DMG1701353 | SRR6346280 | Port Phillip Bay | South Mornington Peninsula | 2015 |
| DMG1701351 | SRR6346282 | Port Phillip Bay | North Mornington Peninsula | 2015 |
| DMG1701517 | SRR6346286 | Gippsland | Gippsland | 1945 |
| DMG1701516 | SRR6346287 | Gippsland | Gippsland | 1945 |
| DMG1701513 | SRR6346290 | Phillip Island | Phillip Island | 1994 |
| DMG1701428 | SRR6346292 | Far East Gippsland | Far East Gippsland | 2002 |
| DMG1701398 | SRR6346296 | Port Phillip Bay | Bellarine Peninsula | 2006 |
| DMG1701397 | SRR6346297 | Gippsland | Gippsland | 2005 |
| DMG1701389 | SRR6346299 | Port Phillip Bay | North Mornington Peninsula | 2016 |
| DMG1701393 | SRR6346303 | Port Phillip Bay | North Mornington Peninsula | 2016 |
| DMG1701396 | SRR6346304 | Port Phillip Bay | North Mornington Peninsula | 2005 |
| DMG1701508 | SRR6346314 | Phillip Island | Phillip Island | 1994 |
| DMG1701512 | SRR6346316 | Phillip Island | Phillip Island | 1993 |
| DMG1701409 | SRR6346328 | Port Phillip Bay | Bellarine Peninsula | 2008 |
| DMG1701403 | SRR6346330 | Port Phillip Bay | North Mornington Peninsula | 2007 |
| DMG1701400 | SRR6346331 | Port Phillip Bay | North Mornington Peninsula | 2015 |
| DMG1701406 | SRR6346333 | Port Phillip Bay | Bellarine Peninsula | 2008 |
| DMG1701404 | SRR6346335 | Port Phillip Bay | North Mornington Peninsula | 2008 |
| DMG1701405 | SRR6346336 | Port Phillip Bay | Bellarine Peninsula | 2008 |
| DMG1701495 | SRR6346339 | Gippsland | Gippsland | 2005 |
| DMG1701493 | SRR6346340 | Far East Gippsland | Far East Gippsland | 2015 |
| DMG1701499 | SRR6346343 | Far East Gippsland | Far East Gippsland | 2005 |
| DMG1701477 | SRR6346351 | Gippsland | Gippsland | 2003 |
| DMG1701377 | SRR6346358 | Port Phillip Bay | South Mornington Peninsula | 2016 |
| DMG1701376 | SRR6346359 | Port Phillip Bay | Bellarine Peninsula | 2016 |
| DMG1701373 | SRR6346362 | Port Phillip Bay | North Mornington Peninsula | 2016 |
| DMG1701371 | SRR6346364 | Port Phillip Bay | Bellarine Peninsula | 2016 |
| DMG1701384 | SRR6346374 | Port Phillip Bay | Bellarine Peninsula | 2016 |
| DMG1701522 | SRR6346377 | Phillip Island | Phillip Island | 2002 |

S1 Table: *M. ulcerans* isolates and their respective sequencing reactions.
