# Supplementary material for: Buruli ulcer in Australia: Evidence for a new endemic focus at Batemans Bay, New South Wales
Source: PLoS Negl Trop Dis. 2024 Dec 13;18(12):e0012702. doi: 10.1371/journal.pntd.0012702 (PMC11676496; doi:10.1371/journal.pntd.0012702)
Supplement: S1 Fig — Shown is the relevant region of the M. ulcerans core-genome phylogeny and the internal tree nodes with divergence time estimations. (DOCX) [file pntd.0012702.s002.docx]

**
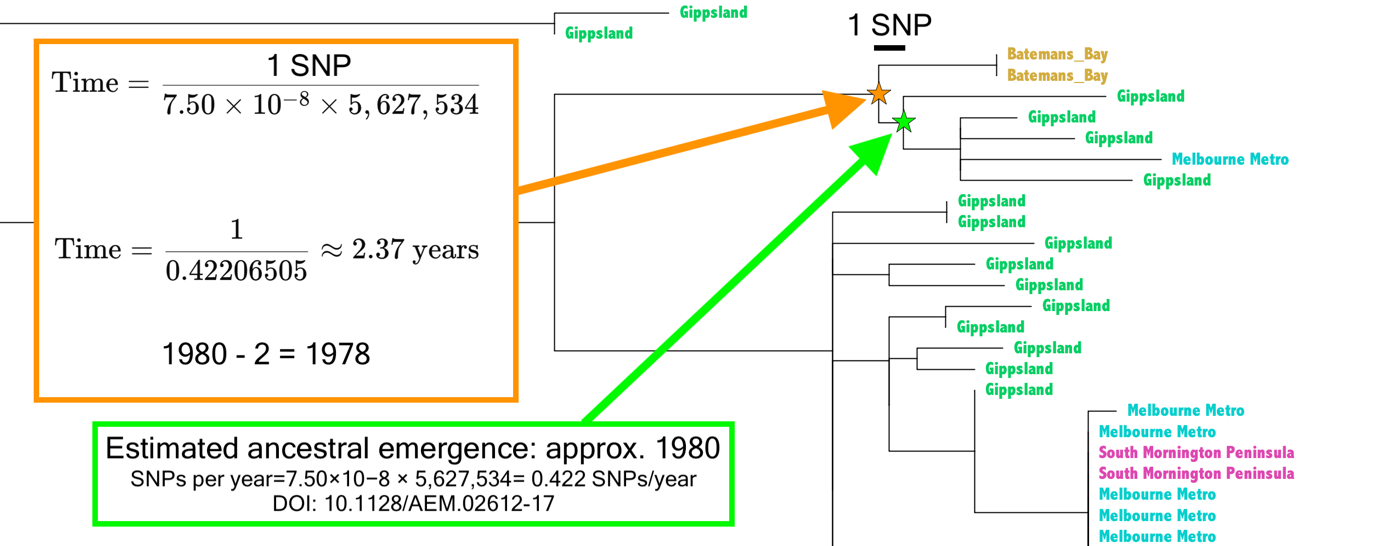
**

S1 FIg: Estimation of the emergence date of the Batemans Bay *M. ulcerans* genotype. Shown is the relevant region of the *M. ulcerans* core-genome phylogeny and the internal tree nodes with divergence time estimations.
